# Supplementary material for: Navigating the biopsychosocial landscape: A systematic review on the association between social support and chronic pain
Source: PLoS One. 2025 Apr 29;20(4):e0321750. doi: 10.1371/journal.pone.0321750 (PMC12040255; doi:10.1371/journal.pone.0321750)
Supplement: S1 Text — The research equation was carried out in six different databases (PubMed, Embase, PsycINFO, Cochrane Library, CINAHL and Scopus). The equations were constructed around three main concepts:” adult”, “chronic pain” and “social support”. Variations between research strings from one database to another are linked to the specificity of the thesaurus of each database. (DOCX) [file pone.0321750.s001.docx]

**S1 File. Research Equations**

**CINAHL**

(MH "Adult" OR MH "Aged" OR MH "Middle Age" OR MH "Young Adult" OR TI "adult" OR TI "adults" OR TI "elderly" OR TI "middle aged" OR AB "adult" OR AB "adults" OR AB "elderly" OR AB "middle aged" ) AND (MH "Chronic pain" OR TI "Chronic Pain" OR TI "Chronic Postoperative Pain*" OR TI "Chronic Post-operative Pain*" OR TI "Chronic Postsurgical Pain*" OR TI "Chronic Post-surgical Pain*" OR TI "chronic posttraumatic pain*" OR TI "chronic post-traumatic pain*" OR TI "fibromyalgia" OR AB "Chronic Pain" OR AB "Chronic Postoperative Pain*" OR AB "Chronic Post-operative Pain*" OR AB "Chronic Postsurgical Pain*" OR AB "Chronic Post-surgical Pain*" OR AB "chronic posttraumatic pain*" OR AB "chronic post-traumatic pain*" OR AB "fibromyalgia" ) AND (MH "Social support" OR TI "appraisal support" OR TI "Informational support" OR TI "Instrumental Support" OR TI "Marital support" OR TI "peer support" OR TI "Perceived Social Supports" OR TI "perceived support" OR TI "received social supports" OR TI "received support" OR TI "social embeddedness" OR TI "Social Support" OR TI "structural support" OR AB "appraisal support" OR AB "Informational support" OR AB "Instrumental Support" OR AB "Marital support" OR AB "peer support" OR AB "Perceived Social Supports" OR AB "perceived support" OR AB "received social supports" OR AB "received support" OR AB "social embeddedness" OR AB "Social Support" OR AB "structural support" )

**COCHRANE**

([mh "adult"] OR [mh "aged"] OR [mh "middle aged"] OR [mh "young adult"] OR ("adult"):ti,ab OR ("adults"):ti,ab OR ("elderly"):ti,ab OR ("middle aged"):ti,ab ) AND ([mh "Chronic Pain"] OR ("Chronic Pain"):ti,ab OR ("Chronic Postoperative Pain*"):ti,ab OR ("Chronic Post-operative Pain*"):ti,ab OR ("Chronic Postsurgical Pain*"):ti,ab OR ("Chronic Post-surgical Pain*"):ti,ab OR ("chronic posttraumatic pain*"):ti,ab OR ("chronic post-traumatic pain*"):ti,ab OR ("fibromyalgia"):ti,ab ) AND ([mh "Social Support"] OR ("appraisal support"):ti,ab OR ("Informational support"):ti,ab OR ("Instrumental Support"):ti,ab OR ("Marital support"):ti,ab OR ("peer support"):ti,ab OR ("Perceived Social Supports"):ti,ab OR ("perceived support"):ti,ab OR ("received social supports"):ti,ab OR ("received support"):ti,ab OR ("social embeddedness"):ti,ab OR ("Social Support"):ti,ab OR ("structural support"):ti,ab )

**EMBASE**

('adult'/de OR 'middle aged'/de OR 'young adult'/de OR 'adult':ti,ab OR 'adults':ti,ab OR 'elderly':ti,ab OR 'Middle Aged':ti,ab ) AND ('chronic pain'/exp OR 'dyspareunia'/de OR 'female genital pain'/exp OR 'genital pain'/exp OR 'perineal pain'/exp OR 'fibromyalgia'/exp OR 'rheumatic polymyalgia'/exp OR 'musculoskeletal pain'/exp OR 'backache'/exp OR 'neuropathic pain'/de OR 'radicular pain'/exp OR 'sciatica'/exp OR 'postoperative pain'/exp OR 'pelvic pain'/exp OR 'phantom pain'/exp OR 'posttraumatic pain'/exp OR 'psychogenic pain'/exp OR 'vagina pain'/exp OR 'vulvodynia'/exp OR 'pain intensity'/exp OR 'pain severity'/exp OR 'back ache':ti,ab OR 'back pain':ti,ab OR 'backache':ti,ab OR 'backpain':ti,ab OR 'Cervical Pain':ti,ab OR 'Cervicalgia':ti,ab OR 'Cervicalgias':ti,ab OR 'Cervicodynia':ti,ab OR 'Chronic Pain':ti,ab OR 'Chronic Postoperative Pain*':ti,ab OR 'Chronic Post-operative Pain*':ti,ab OR 'Chronic Postsurgical Pain*':ti,ab OR 'Chronic Post-surgical Pain*':ti,ab OR 'chronic posttraumatic pain*':ti,ab OR 'chronic post-traumatic pain*':ti,ab OR 'CRPS Type II':ti,ab OR 'deep pain*':ti,ab OR 'dorsalgia*':ti,ab OR 'dyspareunia*':ti,ab OR 'failed back surgery':ti,ab OR 'failed back syndrome':ti,ab OR 'fibromyalgia':ti,ab OR 'Fibromyalgia-Fibromyositis Syndrome':ti,ab OR 'Fibromyalgias':ti,ab OR 'Fibromyositis-Fibromyalgia Syndrome':ti,ab OR 'Fibrositis':ti,ab OR 'forestier certonciny syndrome':ti,ab OR 'genital pain':ti,ab OR 'Intractable Pain':ti,ab OR 'lowback pain':ti,ab OR 'lumbago':ti,ab OR 'lumbal pain':ti,ab OR 'lumbal syndrome':ti,ab OR 'lumbalgesia':ti,ab OR 'lumbalgia':ti,ab OR 'lumbar pain':ti,ab OR 'lumbar spine syndrome':ti,ab OR 'lumbodynia':ti,ab OR 'lumbosacral pain':ti,ab OR 'lumbosacral root syndrome':ti,ab OR 'malignant pain':ti,ab OR 'Muscle Pain':ti,ab OR 'Musculoskeletal Pain':ti,ab OR 'Myalgia':ti,ab OR 'Neck Ache':ti,ab OR 'Neck Pain':ti,ab OR 'Neckache':ti,ab OR 'Neckaches':ti,ab OR 'Nerve Pain':ti,ab OR 'nerve root pain':ti,ab OR 'Neuralgia':ti,ab OR 'Neuropathic Pain':ti,ab OR 'pain disorder':ti,ab OR 'pain syndrome':ti,ab OR 'pelipathia vegetativa':ti,ab OR 'pelvic outlet syndrome':ti,ab OR 'pelvic pain':ti,ab OR 'pelvic syndrome':ti,ab OR 'pelvis pain':ti,ab OR 'pelvis syndrome':ti,ab OR 'perineal pain':ti,ab OR 'perineum pain':ti,ab OR 'Phantom Limb Pain':ti,ab OR 'Phantom Pain':ti,ab OR 'piriform syndrome':ti,ab OR 'piriformis muscle syndrome':ti,ab OR 'Piriformis syndrome':ti,ab OR 'polymyalgia arteritica':ti,ab OR 'polymyalgia rheumatica':ti,ab OR 'post operation pain':ti,ab OR 'post traumatic pain':ti,ab OR 'postlaminectomy syndrome':ti,ab OR 'post-laminectomy syndrome':ti,ab OR 'postoperative pain':ti,ab OR 'posttraumatic pain':ti,ab OR 'post-traumatic pain':ti,ab OR 'pseudopolyarthritis rhizomelica':ti,ab OR 'psychalgia':ti,ab OR 'psychogenic pain':ti,ab OR 'Pudendal Canal Entrapment Syndrome':ti,ab OR 'Pudendal Nerve Entrapment':ti,ab OR 'Pudendal Neuropathies':ti,ab OR 'Pudendal Neuropathy':ti,ab OR 'radiculalgia':ti,ab OR 'radicular pain':ti,ab OR 'Refractory Pain':ti,ab OR 'rheumatic polymyalgia':ti,ab OR 'rheumatism':ti,ab OR 'rhizomelic pseudopolyarthritis':ti,ab OR 'sciatic pain':ti,ab OR 'Sciatica':ti,ab OR 'somatoform pain':ti,ab OR 'treatment related pain':ti,ab OR 'vagina pain':ti,ab OR 'vaginal pain':ti,ab OR 'vulval pain':ti,ab OR 'vulvodynia':ti,ab ) AND ('social support'/exp OR 'caregiver support'/exp OR 'social identity'/exp OR 'social aspects and related phenomena'/de OR 'community participation'/de OR 'community support'/exp OR 'social connectedness'/exp OR 'spouse'/exp OR 'appraisal support':ti,ab OR 'caregiver support':ti,ab OR 'Close Relationships':ti,ab OR 'Community Support':ti,ab OR 'Community Supports':ti,ab OR 'couple support':ti,ab OR 'Couples':ti,ab OR 'emotional care':ti,ab OR 'emotional support':ti,ab OR 'family support':ti,ab OR 'friend support':ti,ab OR 'Group Cohesion':ti,ab OR 'Group Cohesions':ti,ab OR 'Group Cohesiveness':ti,ab OR 'Group Cohesivenesses':ti,ab OR 'Group Solidarities':ti,ab OR 'Group Solidarity':ti,ab OR 'Husband':ti,ab OR 'Husbands':ti,ab OR 'Husband-Wife Communication':ti,ab OR 'Husband-Wife Communications':ti,ab OR 'Informational support':ti,ab OR 'Instrumental Support':ti,ab OR 'Interpersonal Relation':ti,ab OR 'Interpersonal Relations':ti,ab OR 'Interpersonal Relationships':ti,ab OR 'Marital Relations':ti,ab OR 'Marital support':ti,ab OR 'Partner Communication':ti,ab OR 'Partner Responses':ti,ab OR 'peer acceptance':ti,ab OR 'peer counseling':ti,ab OR 'peer group':ti,ab OR 'peer relation':ti,ab OR 'peer relations':ti,ab OR 'peer relationship':ti,ab OR 'peer relationships':ti,ab OR 'peer support':ti,ab OR 'Peer-support':ti,ab OR 'Peers':ti,ab OR 'Perceived Social Supports':ti,ab OR 'perceived support':ti,ab OR 'Psychosocial Support':ti,ab OR 'received social supports':ti,ab OR 'received support':ti,ab OR 'Relationship Quality':ti,ab OR 'Relationship Satisfaction':ti,ab OR 'self-esteem support':ti,ab OR 'significant other support':ti,ab OR 'Significant Others':ti,ab OR 'social aspects and related phenomena':ti,ab OR 'Social Cohesion':ti,ab OR 'Social Cohesions':ti,ab OR 'social cohesiveness':ti,ab OR 'social connectedness':ti,ab OR 'social connection':ti,ab OR 'social connectivity':ti,ab OR social embeddedness:ti,ab OR 'social environment':ti,ab OR 'social factor':ti,ab OR 'social factors':ti,ab OR 'Social Inclusion':ti,ab OR 'social interaction':ti,ab OR 'Social Relationship':ti,ab OR 'Social Relationships':ti,ab OR 'Social Support':ti,ab OR 'social therapy':ti,ab OR 'solicitousness':ti,ab OR 'spousal assisted support':ti,ab OR 'spousal support':ti,ab OR 'Spouse':ti,ab OR 'Spouses':ti,ab OR 'structural support':ti,ab OR 'support group':ti,ab OR 'support groups':ti,ab OR 'Wife':ti,ab OR 'Wives':ti,ab )

**PSYCINFO**

(MJMAINSUBJECT.EXACT( "adult" ) OR MJMAINSUBJECT.EXACT( "aged" ) OR MJMAINSUBJECT.EXACT( "middle aged" ) OR MJMAINSUBJECT.EXACT( "young adult" ) OR TI,AB( "adult" ) OR TI,AB( "adults" ) OR TI,AB( "elderly" ) OR TI,AB( "middle aged" ) ) AND (MESH( "Chronic Pain" ) OR MESH( "Cancer Pain" ) OR MESH( "Musculoskeletal Pain" ) OR MJMAINSUBJECT.EXACT( "fibromyalgia" ) OR MJMAINSUBJECT.EXACT( "Pain, Postoperative" ) OR MJMAINSUBJECT.EXACT( "pelvic pain" ) OR MESH( "Neck Pain" ) OR MJMAINSUBJECT.EXACT( "Neuralgia" ) OR MESH( "Causalgia" ) OR MESH( "Piriformis Muscle Syndrome" ) OR MESH( "Pudendal Neuralgia" ) OR MESH( "Sciatica" ) OR MESH( "Pudendal Neuralgia" ) OR MESH( "Pain, Intractable" ) OR TI,AB( "back ache" ) OR TI,AB( "back pain" ) OR TI,AB( "backache" ) OR TI,AB( "backpain" ) OR TI,AB( "Cervical Pain" ) OR TI,AB( "Cervicalgia" ) OR TI,AB( "Cervicalgias" ) OR TI,AB( "Cervicodynia" ) OR TI,AB( "Chronic Pain" ) OR TI,AB( "Chronic Postoperative Pain*" ) OR TI,AB( "Chronic Post-operative Pain*" ) OR TI,AB( "Chronic Postsurgical Pain*" ) OR TI,AB( "Chronic Post-surgical Pain*" ) OR TI,AB( "chronic posttraumatic pain*" ) OR TI,AB( "chronic post-traumatic pain*" ) OR TI,AB( "CRPS Type II" ) OR TI,AB( "deep pain*" ) OR TI,AB( "dorsalgia*" ) OR TI,AB( "dyspareunia*" ) OR TI,AB( "failed back surgery" ) OR TI,AB( "failed back syndrome" ) OR TI,AB( "fibromyalgia" ) OR TI,AB( "Fibromyalgia-Fibromyositis Syndrome" ) OR TI,AB( "Fibromyalgias" ) OR TI,AB( "Fibromyositis-Fibromyalgia Syndrome" ) OR TI,AB( "Fibrositis" ) OR TI,AB( "forestier certonciny syndrome" ) OR TI,AB( "genital pain" ) OR TI,AB( "Intractable Pain" ) OR TI,AB( "lowback pain" ) OR TI,AB( "lumbago" ) OR TI,AB( "lumbal pain" ) OR TI,AB( "lumbal syndrome" ) OR TI,AB( "lumbalgesia" ) OR TI,AB( "lumbalgia" ) OR TI,AB( "lumbar pain" ) OR TI,AB( "lumbar spine syndrome" ) OR TI,AB( "lumbodynia" ) OR TI,AB( "lumbosacral pain" ) OR TI,AB( "lumbosacral root syndrome" ) OR TI,AB( "malignant pain" ) OR TI,AB( "Muscle Pain" ) OR TI,AB( "Musculoskeletal Pain" ) OR TI,AB( "Myalgia" ) OR TI,AB( "Neck Ache" ) OR TI,AB( "Neck Pain" ) OR TI,AB( "Neckache" ) OR TI,AB( "Neckaches" ) OR TI,AB( "Nerve Pain" ) OR TI,AB( "nerve root pain" ) OR TI,AB( "Neuralgia" ) OR TI,AB( "Neuropathic Pain" ) OR TI,AB( "pain disorder" ) OR TI,AB( "pain syndrome" ) OR TI,AB( "pelipathia vegetativa" ) OR TI,AB( "pelvic outlet syndrome" ) OR TI,AB( "pelvic pain" ) OR TI,AB( "pelvic syndrome" ) OR TI,AB( "pelvis pain" ) OR TI,AB( "pelvis syndrome" ) OR TI,AB( "perineal pain" ) OR TI,AB( "perineum pain" ) OR TI,AB( "Phantom Limb Pain" ) OR TI,AB( "Phantom Pain" ) OR TI,AB( "piriform syndrome" ) OR TI,AB( "piriformis muscle syndrome" ) OR TI,AB( "Piriformis syndrome" ) OR TI,AB( "polymyalgia arteritica" ) OR TI,AB( "polymyalgia rheumatica" ) OR TI,AB( "post operation pain" ) OR TI,AB( "post traumatic pain" ) OR TI,AB( "postlaminectomy syndrome" ) OR TI,AB( "post-laminectomy syndrome" ) OR TI,AB( "postoperative pain" ) OR TI,AB( "posttraumatic pain" ) OR TI,AB( "post-traumatic pain" ) OR TI,AB( "pseudopolyarthritis rhizomelica" ) OR TI,AB( "psychalgia" ) OR TI,AB( "psychogenic pain" ) OR TI,AB( "Pudendal Canal Entrapment Syndrome" ) OR TI,AB( "Pudendal Nerve Entrapment" ) OR TI,AB( "Pudendal Neuropathies" ) OR TI,AB( "Pudendal Neuropathy" ) OR TI,AB( "radiculalgia" ) OR TI,AB( "radicular pain" ) OR TI,AB( "Refractory Pain" ) OR TI,AB( "rheumatic polymyalgia" ) OR TI,AB( "rheumatism" ) OR TI,AB( "rhizomelic pseudopolyarthritis" ) OR TI,AB( "sciatic pain" ) OR TI,AB( "Sciatica" ) OR TI,AB( "somatoform pain" ) OR TI,AB( "treatment related pain" ) OR TI,AB( "vagina pain" ) OR TI,AB( "vaginal pain" ) OR TI,AB( "vulval pain" ) OR TI,AB( "vulvodynia" ) ) AND (MESH( "Social Support" ) OR MESH( "Community Support" ) OR MESH( "Spouses" ) OR MESH( "Social Support Networks" ) OR MESH( "Relationship Satisfaction" ) OR TI,AB( "appraisal support" ) OR TI,AB( "caregiver support" ) OR TI,AB( "Close Relationships" ) OR TI,AB( "Community Support" ) OR TI,AB( "Community Supports" ) OR TI,AB( "couple support" ) OR TI,AB( "Couples" ) OR TI,AB( "emotional care" ) OR TI,AB( "emotional support" ) OR TI,AB( "family support" ) OR TI,AB( "friend support" ) OR TI,AB( "Group Cohesion" ) OR TI,AB( "Group Cohesions" ) OR TI,AB( "Group Cohesiveness" ) OR TI,AB( "Group Cohesivenesses" ) OR TI,AB( "Group Solidarities" ) OR TI,AB( "Group Solidarity" ) OR TI,AB( "Husband" ) OR TI,AB( "Husbands" ) OR TI,AB( "Husband-Wife Communication" ) OR TI,AB( "Husband-Wife Communications" ) OR TI,AB( "Informational support" ) OR TI,AB( "Instrumental Support" ) OR TI,AB( "Interpersonal Relation" ) OR TI,AB( "Interpersonal Relations" ) OR TI,AB( "Interpersonal Relationships" ) OR TI,AB( "Marital Relations" ) OR TI,AB( "Marital support" ) OR TI,AB( "Partner Communication" ) OR TI,AB( "Partner Responses" ) OR TI,AB( "peer acceptance" ) OR TI,AB( "peer counseling" ) OR TI,AB( "peer group" ) OR TI,AB( "peer relation" ) OR TI,AB( "peer relations" ) OR TI,AB( "peer relationship" ) OR TI,AB( "peer relationships" ) OR TI,AB( "peer support" ) OR TI,AB( "Peer-support" ) OR TI,AB( "Peers" ) OR TI,AB( "Perceived Social Supports" ) OR TI,AB( "perceived support" ) OR TI,AB( "Psychosocial Support" ) OR TI,AB( "received social supports" ) OR TI,AB( "received support" ) OR TI,AB( "Relationship Quality" ) OR TI,AB( "Relationship Satisfaction" ) OR TI,AB( "self-esteem support" ) OR TI,AB( "significant other support" ) OR TI,AB( "Significant Others" ) OR TI,AB( "social aspects and related phenomena" ) OR TI,AB( "Social Cohesion" ) OR TI,AB( "Social Cohesions" ) OR TI,AB( "social cohesiveness" ) OR TI,AB( "social connectedness" ) OR TI,AB( "social connection" ) OR TI,AB( "social connectivity" ) OR TI,AB( "social embeddedness" ) OR TI,AB( "social environment" ) OR TI,AB( "social factor" ) OR TI,AB( "social factors" ) OR TI,AB( "Social Inclusion" ) OR TI,AB( "social interaction" ) OR TI,AB( "Social Relationship" ) OR TI,AB( "Social Relationships" ) OR TI,AB( "Social Support" ) OR TI,AB( "social therapy" ) OR TI,AB( "solicitousness" ) OR TI,AB( "spousal assisted support" ) OR TI,AB( "spousal support" ) OR TI,AB( "Spouse" ) OR TI,AB( "Spouses" ) OR TI,AB( "structural support" ) OR TI,AB( "support group" ) OR TI,AB( "support groups" ) OR TI,AB( "Wife" ) OR TI,AB( "Wives" ) )

**PUBMED**

("adult"[mh:NoExp] OR "aged"[mh:NoExp] OR "middle aged"[mh:NoExp] OR "young adult"[mh:NoExp] OR "adult"[tiab] OR "adults"[tiab] OR "elderly"[tiab] OR "Middle Aged"[tiab] ) AND ("Chronic Pain"[mh] OR "Cancer Pain"[mh] OR "Musculoskeletal Pain"[mh] OR "fibromyalgia"[mh:NoExp] OR "Pain, Postoperative"[mh:NoExp] OR "pelvic pain"[mh:NoExp] OR "Neck Pain"[mh] OR "Neuralgia"[mh:NoExp] OR "Causalgia"[mh] OR "Piriformis Muscle Syndrome"[mh] OR "Pudendal Neuralgia"[mh] OR "Sciatica"[mh] OR "Pudendal Neuralgia"[mh] OR "Pain, Intractable"[mh] OR "back ache"[tiab] OR "back pain"[tiab] OR "backache"[tiab] OR "backpain"[tiab] OR "Cervical Pain"[tiab] OR "Cervicalgia"[tiab] OR "Cervicalgias"[tiab] OR "Cervicodynia"[tiab] OR "Chronic Pain"[tiab] OR "Chronic Postoperative Pain*"[tiab] OR "Chronic Post-operative Pain*"[tiab] OR "Chronic Postsurgical Pain*"[tiab] OR "Chronic Post-surgical Pain*"[tiab] OR "chronic posttraumatic pain*"[tiab] OR "chronic post-traumatic pain*"[tiab] OR "CRPS Type II"[tiab] OR "deep pain*"[tiab] OR "dorsalgia*"[tiab] OR "dyspareunia*"[tiab] OR "failed back surgery"[tiab] OR "failed back syndrome"[tiab] OR "fibromyalgia"[tiab] OR "Fibromyalgia-Fibromyositis Syndrome"[tiab] OR "Fibromyalgias"[tiab] OR "Fibromyositis-Fibromyalgia Syndrome"[tiab] OR "Fibrositis"[tiab] OR "forestier certonciny syndrome"[tiab] OR "genital pain"[tiab] OR "Intractable Pain"[tiab] OR "lowback pain"[tiab] OR "lumbago"[tiab] OR "lumbal pain"[tiab] OR "lumbal syndrome"[tiab] OR "lumbalgesia"[tiab] OR "lumbalgia"[tiab] OR "lumbar pain"[tiab] OR "lumbar spine syndrome"[tiab] OR "lumbodynia"[tiab] OR "lumbosacral pain"[tiab] OR "lumbosacral root syndrome"[tiab] OR "malignant pain"[tiab] OR "Muscle Pain"[tiab] OR "Musculoskeletal Pain"[tiab] OR "Myalgia"[tiab] OR "Neck Ache"[tiab] OR "Neck Pain"[tiab] OR "Neckache"[tiab] OR "Neckaches"[tiab] OR "Nerve Pain"[tiab] OR "nerve root pain"[tiab] OR "Neuralgia"[tiab] OR "Neuropathic Pain"[tiab] OR "pain disorder"[tiab] OR "pain syndrome"[tiab] OR "pelipathia vegetativa"[tiab] OR "pelvic outlet syndrome"[tiab] OR "pelvic pain"[tiab] OR "pelvic syndrome"[tiab] OR "pelvis pain"[tiab] OR "pelvis syndrome"[tiab] OR "perineal pain"[tiab] OR "perineum pain"[tiab] OR "Phantom Limb Pain"[tiab] OR "Phantom Pain"[tiab] OR "piriform syndrome"[tiab] OR "piriformis muscle syndrome"[tiab] OR "Piriformis syndrome"[tiab] OR "polymyalgia arteritica"[tiab] OR "polymyalgia rheumatica"[tiab] OR "post operation pain"[tiab] OR "post traumatic pain"[tiab] OR "postlaminectomy syndrome"[tiab] OR "post-laminectomy syndrome"[tiab] OR "postoperative pain"[tiab] OR "posttraumatic pain"[tiab] OR "post-traumatic pain"[tiab] OR "pseudopolyarthritis rhizomelica"[tiab] OR "psychalgia"[tiab] OR "psychogenic pain"[tiab] OR "Pudendal Canal Entrapment Syndrome"[tiab] OR "Pudendal Nerve Entrapment"[tiab] OR "Pudendal Neuropathies"[tiab] OR "Pudendal Neuropathy"[tiab] OR "radiculalgia"[tiab] OR "radicular pain"[tiab] OR "Refractory Pain"[tiab] OR "rheumatic polymyalgia"[tiab] OR "rheumatism"[tiab] OR "rhizomelic pseudopolyarthritis"[tiab] OR "sciatic pain"[tiab] OR "Sciatica"[tiab] OR "somatoform pain"[tiab] OR "treatment related pain"[tiab] OR "vagina pain"[tiab] OR "vaginal pain"[tiab] OR "vulval pain"[tiab] OR "vulvodynia"[tiab] ) AND ("Social Support"[mh] OR "Community Support"[mh] OR "Interpersonal Relations"[mh:NoExp] OR "Social Cohesion"[mh] OR "Spouses"[mh] OR "appraisal support"[tiab] OR "caregiver support"[tiab] OR "Close Relationships"[tiab] OR "Community Support"[tiab] OR "Community Supports"[tiab] OR "couple support"[tiab] OR "Couples"[tiab] OR "emotional care"[tiab] OR "emotional support"[tiab] OR "family support"[tiab] OR "friend support"[tiab] OR "Group Cohesion"[tiab] OR "Group Cohesions"[tiab] OR "Group Cohesiveness"[tiab] OR "Group Cohesivenesses"[tiab] OR "Group Solidarities"[tiab] OR "Group Solidarity"[tiab] OR "Husband"[tiab] OR "Husbands"[tiab] OR "Husband-Wife Communication"[tiab] OR "Husband-Wife Communications"[tiab] OR "Informational support"[tiab] OR "Instrumental Support"[tiab] OR "Interpersonal Relation"[tiab] OR "Interpersonal Relations"[tiab] OR "Interpersonal Relationships"[tiab] OR "Marital Relations"[tiab] OR "Marital support"[tiab] OR "Partner Communication"[tiab] OR "Partner Responses"[tiab] OR "peer acceptance"[tiab] OR "peer counseling"[tiab] OR "peer group"[tiab] OR "peer relation"[tiab] OR "peer relations"[tiab] OR "peer relationship"[tiab] OR "peer relationships"[tiab] OR "peer support"[tiab] OR "Peer-support"[tiab] OR "Peers"[tiab] OR "Perceived Social Supports"[tiab] OR "perceived support"[tiab] OR "Psychosocial Support"[tiab] OR "received social supports"[tiab] OR "received support"[tiab] OR "Relationship Quality"[tiab] OR "Relationship Satisfaction"[tiab] OR "self-esteem support"[tiab] OR "significant other support"[tiab] OR "Significant Others"[tiab] OR "social aspects and related phenomena"[tiab] OR "Social Cohesion"[tiab] OR "Social Cohesions"[tiab] OR "social cohesiveness"[tiab] OR "social connectedness"[tiab] OR "social connection"[tiab] OR "social connectivity"[tiab] OR "social embeddedness"[tiab] OR "social environment"[tiab] OR "social factor"[tiab] OR "social factors"[tiab] OR "Social Inclusion"[tiab] OR "social interaction"[tiab] OR "Social Relationship"[tiab] OR "Social Relationships"[tiab] OR "Social Support"[tiab] OR "social therapy"[tiab] OR "solicitousness"[tiab] OR "spousal assisted support"[tiab] OR "spousal support"[tiab] OR "Spouse"[tiab] OR "Spouses"[tiab] OR "structural support"[tiab] OR "support group"[tiab] OR "support groups"[tiab] OR "Wife"[tiab] OR "Wives"[tiab] OR "support group"[tiab] OR "support groups"[tiab] OR "Wife"[tiab] OR "Wives"[tiab] )

**SCOPUS**

(KEY ( {adult} ) OR KEY ( {aged} ) OR KEY ( {middle aged} ) OR KEY ( {young adult} ) OR TITLE-ABS ( {adult} ) OR TITLE-ABS ( {adults} ) OR TITLE-ABS ( {elderly} ) OR TITLE-ABS ( {middle aged} ) ) AND (KEY ( {Chronic Pain} ) OR KEY ( {Cancer Pain} ) OR KEY ( {Musculoskeletal Pain} ) OR KEY ( {fibromyalgia} ) OR KEY ( {Pain, Postoperative} ) OR KEY ( {pelvic pain} ) OR KEY ( {pain intensity} ) OR KEY ( {pain severity} ) OR KEY ( {Neck Pain} ) OR KEY ( {Neuralgia} ) OR KEY ( {Causalgia} ) OR KEY ( {Piriformis Muscle Syndrome} ) OR KEY ( {Pudendal Neuralgia} ) OR KEY ( {Sciatica} ) OR KEY ( {Pudendal Neuralgia} ) OR KEY ( {Pain, Intractable} ) OR TITLE-ABS ( {back ache} ) OR TITLE-ABS ( {back pain} ) OR TITLE-ABS ( {backache} ) OR TITLE-ABS ( {backpain} ) OR TITLE-ABS ( {Cervical Pain} ) OR TITLE-ABS ( {Cervicalgia} ) OR TITLE-ABS ( {Cervicalgias} ) OR TITLE-ABS ( {Cervicodynia} ) OR TITLE-ABS ( {Chronic Pain} ) OR TITLE-ABS ( {Chronic Postoperative Pain*} ) OR TITLE-ABS ( {Chronic Post-operative Pain*} ) OR TITLE-ABS ( {Chronic Postsurgical Pain*} ) OR TITLE-ABS ( {Chronic Post-surgical Pain*} ) OR TITLE-ABS ( {chronic posttraumatic pain*} ) OR TITLE-ABS ( {chronic post-traumatic pain*} ) OR TITLE-ABS ( {CRPS Type II} ) OR TITLE-ABS ( {deep pain*} ) OR TITLE-ABS ( {dorsalgia*} ) OR TITLE-ABS ( {dyspareunia*} ) OR TITLE-ABS ( {failed back surgery} ) OR TITLE-ABS ( {failed back syndrome} ) OR TITLE-ABS ( {fibromyalgia} ) OR TITLE-ABS ( {Fibromyalgia-Fibromyositis Syndrome} ) OR TITLE-ABS ( {Fibromyalgias} ) OR TITLE-ABS ( {Fibromyositis-Fibromyalgia Syndrome} ) OR TITLE-ABS ( {Fibrositis} ) OR TITLE-ABS ( {forestier certonciny syndrome} ) OR TITLE-ABS ( {genital pain} ) OR TITLE-ABS ( {Intractable Pain} ) OR TITLE-ABS ( {lowback pain} ) OR TITLE-ABS ( {lumbago} ) OR TITLE-ABS ( {lumbal pain} ) OR TITLE-ABS ( {lumbal syndrome} ) OR TITLE-ABS ( {lumbalgesia} ) OR TITLE-ABS ( {lumbalgia} ) OR TITLE-ABS ( {lumbar pain} ) OR TITLE-ABS ( {lumbar spine syndrome} ) OR TITLE-ABS ( {lumbodynia} ) OR TITLE-ABS ( {lumbosacral pain} ) OR TITLE-ABS ( {lumbosacral root syndrome} ) OR TITLE-ABS ( {malignant pain} ) OR TITLE-ABS ( {Muscle Pain} ) OR TITLE-ABS ( {Musculoskeletal Pain} ) OR TITLE-ABS ( {Myalgia} ) OR TITLE-ABS ( {Neck Ache} ) OR TITLE-ABS ( {Neck Pain} ) OR TITLE-ABS ( {Neckache} ) OR TITLE-ABS ( {Neckaches} ) OR TITLE-ABS ( {Nerve Pain} ) OR TITLE-ABS ( {nerve root pain} ) OR TITLE-ABS ( {Neuralgia} ) OR TITLE-ABS ( {Neuropathic Pain} ) OR TITLE-ABS ( {pain disorder} ) OR TITLE-ABS ( {pain syndrome} ) OR TITLE-ABS ( {pelipathia vegetativa} ) OR TITLE-ABS ( {pelvic outlet syndrome} ) OR TITLE-ABS ( {pelvic pain} ) OR TITLE-ABS ( {pelvic syndrome} ) OR TITLE-ABS ( {pelvis pain} ) OR TITLE-ABS ( {pelvis syndrome} ) OR TITLE-ABS ( {perineal pain} ) OR TITLE-ABS ( {perineum pain} ) OR TITLE-ABS ( {Phantom Limb Pain} ) OR TITLE-ABS ( {Phantom Pain} ) OR TITLE-ABS ( {piriform syndrome} ) OR TITLE-ABS ( {piriformis muscle syndrome} ) OR TITLE-ABS ( {Piriformis syndrome} ) OR TITLE-ABS ( {polymyalgia arteritica} ) OR TITLE-ABS ( {polymyalgia rheumatica} ) OR TITLE-ABS ( {post operation pain} ) OR TITLE-ABS ( {post traumatic pain} ) OR TITLE-ABS ( {postlaminectomy syndrome} ) OR TITLE-ABS ( {post-laminectomy syndrome} ) OR TITLE-ABS ( {postoperative pain} ) OR TITLE-ABS ( {posttraumatic pain} ) OR TITLE-ABS ( {post-traumatic pain} ) OR TITLE-ABS ( {pseudopolyarthritis rhizomelica} ) OR TITLE-ABS ( {psychalgia} ) OR TITLE-ABS ( {psychogenic pain} ) OR TITLE-ABS ( {Pudendal Canal Entrapment Syndrome} ) OR TITLE-ABS ( {Pudendal Nerve Entrapment} ) OR TITLE-ABS ( {Pudendal Neuropathies} ) OR TITLE-ABS ( {Pudendal Neuropathy} ) OR TITLE-ABS ( {radiculalgia} ) OR TITLE-ABS ( {radicular pain} ) OR TITLE-ABS ( {Refractory Pain} ) OR TITLE-ABS ( {rheumatic polymyalgia} ) OR TITLE-ABS ( {rheumatism} ) OR TITLE-ABS ( {rhizomelic pseudopolyarthritis} ) OR TITLE-ABS ( {sciatic pain} ) OR TITLE-ABS ( {Sciatica} ) OR TITLE-ABS ( {somatoform pain} ) OR TITLE-ABS ( {treatment related pain} ) OR TITLE-ABS ( {vagina pain} ) OR TITLE-ABS ( {vaginal pain} ) OR TITLE-ABS ( {vulval pain} ) OR TITLE-ABS ( {vulvodynia} ) ) AND (KEY ( {Social Support} ) OR KEY ( {Social Adjustment} ) OR KEY ( {Social Isolation} ) OR KEY ( {Community Support} ) OR KEY ( {Social Interaction} ) OR KEY ( {Interpersonal Relations} ) OR KEY ( {Social Cohesion} ) OR KEY ( {Spouses} ) OR TITLE-ABS ( {appraisal support} ) OR TITLE-ABS ( {caregiver support} ) OR TITLE-ABS ( {Close Relationships} ) OR TITLE-ABS ( {Community Support} ) OR TITLE-ABS ( {Community Supports} ) OR TITLE-ABS ( {couple support} ) OR TITLE-ABS ( {Couples} ) OR TITLE-ABS ( {emotional care} ) OR TITLE-ABS ( {emotional support} ) OR TITLE-ABS ( {family support} ) OR TITLE-ABS ( {friend support} ) OR TITLE-ABS ( {Group Cohesion} ) OR TITLE-ABS ( {Group Cohesions} ) OR TITLE-ABS ( {Group Cohesiveness} ) OR TITLE-ABS ( {Group Cohesivenesses} ) OR TITLE-ABS ( {Group Solidarities} ) OR TITLE-ABS ( {Group Solidarity} ) OR TITLE-ABS ( {Husband} ) OR TITLE-ABS ( {Husbands} ) OR TITLE-ABS ( {Husband-Wife Communication} ) OR TITLE-ABS ( {Husband-Wife Communications} ) OR TITLE-ABS ( {Informational support} ) OR TITLE-ABS ( {Instrumental Support} ) OR TITLE-ABS ( {Interpersonal Relation} ) OR TITLE-ABS ( {Interpersonal Relations} ) OR TITLE-ABS ( {Interpersonal Relationships} ) OR TITLE-ABS ( {Marital Relations} ) OR TITLE-ABS ( {Marital support} ) OR TITLE-ABS ( {Partner Communication} ) OR TITLE-ABS ( {Partner Responses} ) OR TITLE-ABS ( {peer acceptance} ) OR TITLE-ABS ( {peer counseling} ) OR TITLE-ABS ( {peer group} ) OR TITLE-ABS ( {peer relation} ) OR TITLE-ABS ( {peer relations} ) OR TITLE-ABS ( {peer relationship} ) OR TITLE-ABS ( {peer relationships} ) OR TITLE-ABS ( {peer support} ) OR TITLE-ABS ( {Peer-support} ) OR TITLE-ABS ( {Peers} ) OR TITLE-ABS ( {Perceived Social Supports} ) OR TITLE-ABS ( {perceived support} ) OR TITLE-ABS ( {Psychosocial Support} ) OR TITLE-ABS ( {received social supports} ) OR TITLE-ABS ( {received support} ) OR TITLE-ABS ( {Relationship Quality} ) OR TITLE-ABS ( {Relationship Satisfaction} ) OR TITLE-ABS ( {self-esteem support} ) OR TITLE-ABS ( {significant other support} ) OR TITLE-ABS ( {Significant Others} ) OR TITLE-ABS ( {social aspects and related phenomena} ) OR TITLE-ABS ( {Social Cohesion} ) OR TITLE-ABS ( {Social Cohesions} ) OR TITLE-ABS ( {social cohesiveness} ) OR TITLE-ABS ( {social connectedness} ) OR TITLE-ABS ( {social connection} ) OR TITLE-ABS ( {social connectivity} ) OR TITLE-ABS ( {social embeddedness} ) OR TITLE-ABS ( {social environment} ) OR TITLE-ABS ( {social factor} ) OR TITLE-ABS ( {social factors} ) OR TITLE-ABS ( {Social Inclusion} ) OR TITLE-ABS ( {social interaction} ) OR TITLE-ABS ( {Social Relationship} ) OR TITLE-ABS ( {Social Relationships} ) OR TITLE-ABS ( {Social Support} ) OR TITLE-ABS ( {social therapy} ) OR TITLE-ABS ( {solicitousness} ) OR TITLE-ABS ( {spousal assisted support} ) OR TITLE-ABS ( {spousal support} ) OR TITLE-ABS ( {Spouse} ) OR TITLE-ABS ( {Spouses} ) OR TITLE-ABS ( {structural support} ) OR TITLE-ABS ( {support group} ) OR TITLE-ABS ( {support groups} ) OR TITLE-ABS ( {Wife} ) OR TITLE-ABS ( {Wives} ) )
