# Supplementary material for: Navigating the biopsychosocial landscape: A systematic review on the association between social support and chronic pain
Source: PLoS One. 2025 Apr 29;20(4):e0321750. doi: 10.1371/journal.pone.0321750 (PMC12040255; doi:10.1371/journal.pone.0321750)
Supplement: S2 Text — (DOCX) [file pone.0321750.s002.docx]

**S2 File. Adapted version of the “JBI Critical Appraisal Checklist for Analytical Cross-Sectional Studies**

| Major Components | Response options |  |  |  |
| --- | --- | --- | --- | --- |
| 1. Were the criteria for inclusion in the sample clearly defined? | Yes | No | Unclear | Not applicable |
| 2. Were the study subjects and the setting described in detail? | Yes | No | Unclear | Not applicable |
| 3. Were explicit, standard criteria used for measurement of the condition? | Yes | No | Unclear | Not applicable |
| 4. Were the outcomes measured in a valid and reliable way? | Yes | No | Unclear | Not applicable |
| 5. Was appropriate statistical analysis used? | Yes | No | Unclear | Not applicable |
| Overall appraisal: Include □ Exclude □ Seek further info □ |  |  |  |  |

**Analytical cross sectional studies Critical Appraisal Tool**

Answers: Yes, No, Unclear or Not/Applicable

## Were the criteria for inclusion in the sample clearly defined?

The authors should provide clear inclusion and exclusion criteria that they developed prior to recruitment of the study participants. The inclusion/exclusion criteria should be specified (e.g., risk, stage of disease progression) with sufficient detail and all the necessary information critical to the study.

## Were the study subjects and the setting described in detail?

The study sample should be described in sufficient detail so that other researchers can determine if it is comparable to the population of interest to them. The authors should provide a clear description of the population from which the study participants were selected or recruited, including demographics, location, and time period.

## Were explicit, standard criteria used for measurement of the condition?

It is useful to determine if patients were included in the study based on either a specified diagnosis or definition. This is more likely to decrease the risk of bias. Characteristics are another useful approach to matching groups, and studies that did not use specified diagnostic methods or definitions should provide evidence on matching by key characteristics

## Were the outcomes measured in a valid and reliable way?

Read the methods section of the paper. If for e.g. lung cancer is assessed based on existing definitions or diagnostic criteria, then the answer to this question is likely to be yes. If lung cancer is assessed using observer reported, or self-reported scales, the risk of over- or under-reporting is increased, and objectivity is compromised. Importantly, determine if the measurement tools used were validated instruments as this has a significant impact on outcome assessment validity.

Having established the objectivity of the outcome measurement (e.g. lung cancer) instrument, it’s important to establish how the measurement was conducted. Were those involved in collecting data trained or educated in the use of the instrument/s? (e.g. radiographers). If there was more than one data collector, were they similar in terms of level of education, clinical or research experience, or level of responsibility in the piece of research being appraised?

## Was appropriate statistical analysis used?

As with any consideration of statistical analysis, consideration should be given to whether there was a more appropriate alternate statistical method that could have been used. The methods section should be detailed enough for reviewers to identify which analytical techniques were used (in particular, regression or stratification) and how specific confounders were measured.

For studies utilizing regression analysis, it is useful to identify if the study identified which variables were included and how they related to the outcome. If stratification was the analytical approach used, were the strata of analysis defined by the specified variables? Additionally, it is also important to assess the appropriateness of the analytical strategy in terms of the assumptions associated with the approach as differing methods of analysis are based on differing assumptions about the data and how it will respond.
