# Supplementary material for: Navigating the biopsychosocial landscape: A systematic review on the association between social support and chronic pain
Source: PLoS One. 2025 Apr 29;20(4):e0321750. doi: 10.1371/journal.pone.0321750 (PMC12040255; doi:10.1371/journal.pone.0321750)
Supplement: S3 Text — (DOCX) [file pone.0321750.s003.docx]

**S3 File. Adapted version of the “Quality Assessment for the Systematic Review of Qualitative Evidence**

The quality assessment tool used for the qualitative studies was drawn directly from Appendix D of Hawker et al. This tool contains nine questions, each of which can be answered ‘good’, ‘fair’, ‘poor’ or ‘very poor’. Having applied the tool to the studies, we converted it into a numerical score by assigning the answers from 1 point (very poor) to 4 points (good). This produced a score for each study of a minimum of 9 points and a maximum of 36 points. To create the overall quality grades we used the following definitions: high quality (A), 30–36 points; medium quality (B), 24–29 points; low quality (C), 9–24 points. The nine questions in the tool are as follows:

1. **Abstract and title**. Did they provide a clear description of the study?

**Good**: structured abstract with full information and clear title. **Fair**: abstract with most of the information. **Poor**: inadequate abstract. **Very poor**: no abstract.

1. **Introduction and aims**. Was there a good background section and clear statement of the aims of the research?

**Good**: full but concise background to discussion/study containing up-to-date literature review and highlighting gaps in knowledge; clear statement of aim AND objectives including research questions. **Fair**: some background and literature review; research questions outlined. **Poor**: some background but no aim/objectives/questions OR aims/objectives but inadequate background. **Very poor**: no mention of aims/objectives; no background or literature review.

1. **Method and data**. Is the method appropriate and clearly explained?

**Good**: method is appropriate and described clearly (e.g. questionnaires included); clear details of the data collection. **Fair**: method appropriate, description could be better; data described. **Poor**: questionable whether method is appropriate; method described inadequately; little description of data. **Very poor**: no mention of method AND/OR method inappropriate AND/OR no details of data.

1. **Sampling**. Was the sampling strategy appropriate to address the aims?

**Good**: details (age/gender/race/context) of who was studied and how they were recruited and why this group was targeted; the sample size was justified for the study; response rates shown and explained. **Fair**: sample size justified; most information given but some missing. **Poor**: sampling mentioned but few descriptive details. **Very poor**: no details of sample.

1. **Data analysis**. Was the description of the data analysis sufficiently rigorous?

**Good**: clear description of how analysis was carried out. **Fair**: descriptive discussion of analysis. **Poor**: minimal details about analysis. **Very poor**: no discussion of analysis.

1. **Ethics and bias/limitations**. Has ethical approval been gained and are biases and limitations discussed?

**Good**: ethics: when necessary, issues of confidentiality, sensitivity and consent were addressed; bias: researcher was reflexive and/or aware of study biases and limitations. **Fair**: lip service was paid to above (i.e. these issues were acknowledged). **Poor**: brief mention of issues. **Very poor**: no mention of issues.

1. **Results**. Is there a clear statement of the findings?

**Good**: findings explicit, easy to understand and in logical progression; tables, if present, are explained in text; results relate directly to aims; sufficient data are presented to support findings. **Fair**: findings mentioned but more explanation could be given; data presented relate directly to results. **Poor**: findings presented haphazardly, not explained and do not progress logically from results. **Very poor**: findings not mentioned or do not relate to aims.

1. **Transferability or generalisability**. Are the findings of this study transferable (generalisable) to a wider population?

**Good**: context and setting of the study are described sufficiently to allow comparison with other contexts and settings, plus high score in Q4 (sampling). **Fair**: some context and setting described but more needed to replicate or compare the study with others, plus fair score or higher in Q4. **Poor**: minimal description of context/setting. **Very poor**: no description of context/setting.

1. **Implications and usefulness**. How important are these findings to policy and practice?

**Good**: contributes something new and/or different in terms of understanding/insight or perspective; suggests ideas for further research; suggests implications for policy and/or practice. **Fair**: two of the above. **Poor**: only one of the above. **Very poor**: none of the above.
