# Supplementary material for: Navigating the biopsychosocial landscape: A systematic review on the association between social support and chronic pain
Source: PLoS One. 2025 Apr 29;20(4):e0321750. doi: 10.1371/journal.pone.0321750 (PMC12040255; doi:10.1371/journal.pone.0321750)
Supplement: S1 Table — 2011. In the vote count of statistically significant associations, we decided to give more weight to significant relationships. Therefore correlations count as one, while relationships count as two. (DOCX) [file pone.0321750.s004.docx]

**S1 Table. Adapted level of evidence from Cambell et al. EJP. 2011**

| **Level of evidence** | |
| --- | --- |
| Strong | Consistent associations or lack of found in at least 75% of the high quality and low to moderate RoB studies (minimum of four studies evaluating the outcome) |
| Weak | Consistent associations or lack of found in at least 60% of the high quality and low to moderate RoB studies (minimum of four studies evaluating the outcome) |
| Inconsistent | Consistent associations or lack of found in less than 60% of the high quality and low to moderate RoB studies (minimum of four studies evaluating the outcome) |
| Insufficient | Less than 4 the high quality and low to moderate RoB studies evaluating the outcome |
