# Supplementary material for: Navigating the biopsychosocial landscape: A systematic review on the association between social support and chronic pain
Source: PLoS One. 2025 Apr 29;20(4):e0321750. doi: 10.1371/journal.pone.0321750 (PMC12040255; doi:10.1371/journal.pone.0321750)
Supplement: S2 Table — (DOCX) [file pone.0321750.s005.docx]

**S2 Table. Excluded full-text articles**

| Name of the article | Authors | Year of publication | Journal | Reason for exclusion |
| --- | --- | --- | --- | --- |
| Support, Health, and Fibromyalgia | ? | 1999 | Cochrane Library | Not retrievable |
| Somatization is associated with worse outcome in a chiropractic patient population with neck pain and low back pain. | Ailliet L, et al. | 2016 | Manual therapy - Volume 21, Issue , pp. 170-6 | Outcomes not evaluated |
| Pain in the Time of Corona: Impact of COVID 19 Outbreak on Fibromyalgia Patients | Aloush V,.et al. | 2020 | Arthritis Rheum. - Volume 72, Issue 0, pp. 69-70 | Conference abstract |
| Physical and mental impact of COVID-19 outbreak on fibromyalgia patients | Aloush V,.et al. | 2021 | Clinical and Experimental Rheumatology - Volume 39, Issue 3, pp. S108-S114 | Not chronic pain population |
| Changes in and predictors of pain characteristics in patients with head and neck cancer undergoing radiotherapy | Astrup G.L. et al. | 2015 | Pain - Volume 156, Issue 5, pp. 967-979 | Not chronic pain population |
| Addressing Social Isolation and Social Support in Integrative Group Visits for Chronic Pain | Bahl S. et al. | 2022 | Global Adv. Health Med. - Volume 11, Issue 0, pp. 34 | Conference abstract |
| Predictors of a health promoting lifestyle in women with fibromyalgia syndrome | Beal C.C. et al. | 2009 | Psychology, Health and Medicine - Volume 14, Issue 3, pp. 343-353 | Outcomes not evaluated |
| Chronic Vulvovaginal Pain in Patients of Color: Benefits of Partner Supportiveness in Relation to Sexual Dissatisfaction and Distress | Bennett-Brown M. et al. | 2022 | International Journal of Environmental Research and Public Health - Volume 19, Issue 7, pp. | Outcomes not evaluated |
| Perceived social support in patients with chronic pain with and without opioid use disorder and role of medication for opioid use disorder. | Benville JR. et al. | 2021 | Drug and alcohol dependence - Volume 221, Issue , pp. 108619 | Data could not be extracted |
| Psychosocial aspects of chronic widespread pain and fibromyalgia | Bergman S. et al. | 2005 | Disabil. Rehabil. - Volume 27, Issue 12, pp. 675-683 | Outcomes not evaluated |
| Social support for functional dependence, activity patterns, and chronic pain outcomes: A cross-lagged mediation panel study. | Bernardes S.F. et al. | 2024 | Health psychology : official journal of the Division of Health Psychology, American Psychological Association - Volume , Issue , pp. | Not retrievable |
| Social health in young women with chronic pain. | Boggero I.A. et al. | 2024 | Pain reports - Volume 9, Issue 2, pp. e1146 | Outcomes not evaluated |
| Associations Between Adult Attachment, Pain Catastrophizing, Psychological Inflexibility and Disability in Adults with Chronic Pain. | Borthwick C. et al. | 2024 | Journal of clinical psychology in medical settings - Volume , Issue , pp. | Not chronic pain population |
| Psychosocial resources and chronic pain in individuals with spinal cord injury: evidence from the second Swiss national community survey | Braunwalder C,. et al. | 2021 | Spinal Cord - Volume 59, Issue 4, pp. 410-418 | Not chronic pain population |
| Social functioning of young adults diagnosed with juvenile-onset fibromyalgia syndrome compared to healthy controls: A six-year follow-up | Bromberg M.H. et al. | 2014 | Pain Res. Manage. - Volume 19, Issue 3, pp. e65 | Conference abstract |
| I get by with a little help from my friends: The interaction of chronic pain and organizational support on performance | Byrne Z.S. et al. | 2006 | Journal of Occupational Health Psychology - Volume 11, Issue 3, pp. 215-227 | Not chronic pain population |
| Living with disabling chronic pain: Results from a face-to-face cross-sectional population-based study | Cabrera-León A. | 2018 | BMJ Open - Volume 8, Issue 11, pp. | Not chronic pain population |
| The role of relationship quality and perceived partner responses with pain and disability in those with back pain. | Campbell P. | 2012 | Pain medicine (Malden, Mass.) - Volume 13, Issue 2, pp. 204-14 | Not chronic pain population |
| Perceived Stress, Perceived Social Support, and Global Health in Adults with Chronic Pain. | Castarlenas E. et al. | 2023 | International journal of behavioral medicine - Volume , Issue , pp. | Outcomes not evaluated |
| The impact of psychosocial factors on low back pain: longitudinal results from the Belstress study. | Clays E. | 2007 | Spine (03622436) - Volume 32, Issue 2, pp. 262-268 | Not chronic pain population |
| Correlates of social support in older American Indians: the Native Elder Care Study. | Conte K.P. et al. | 2015 | Aging & Mental Health - Volume 19, Issue 9, pp. 835-843 | Not chronic pain population |
| Chronic pain in traumatized refugees. | Dahl S. et al. | 2006 | Tidsskrift for den Norske laegeforening : tidsskrift for praktisk medicin, ny raekke - Volume 126, Issue 5, pp. 608-10 | Outcomes not evaluated |
| Psychological stress and the fibrositis/fibromyalgia syndrome | Dailey P.A. et al. | 1990 | J. RHEUMATOL. - Volume 17, Issue 10, pp. 1380-1385 | Not retrievable |
| Chronic pain assessment: A seven-factor model | Davidson M.A. et al. | 2008 | Pain Research and Management - Volume 13, Issue 4, pp. 299-308 | Outcomes not evaluated |
| Social support, social connectedness, depressive symptoms and chronic pain symptoms among older adults: A cross-sectional study | Davis M.E. et al. | 2018 | J. Am. Geriatr. Soc. - Volume 66, Issue 0, pp. S156 | Conference Abstract |
| The associations between pain intensity, psychosocial variables, and pain duration/recurrence in a large sample of persons with nonspecific spinal pain | Demmelmaier I. et al | 2008 | Clinical Journal of Pain - Volume 24, Issue 7, pp. 611-619 | Data could not be extracted |
| A Pilot Investigation of Nonpharmacological Pain Management Intervention Groups in Methadone Maintenance Treatment | Dimeola K.A. et al. | 2022 | Journal of Addiction Medicine - Volume 16, Issue 2, pp. 229-234 | Outcomes not evaluated |
| How are socio-demographic and psycho-social factors associated with the prevalence and chronicity of severe pain in 14 different body sites? A cross-sectional population-based survey. | Dorner T.E. et al. | 2018 | Wiener klinische Wochenschrift - Volume 130, Issue 1, pp. 14-22 | Outcomes not evaluated |
| Psychosocial predictors of functional change in recently diagnosed rheumatoid arthritis patients | Evers A. et al. | 1998 | Behaviour Research and Therapy - Volume 36, Issue 2, pp. 179-193 | Not chronic pain population |
| Occupational chronic neck and shoulder pain: study conducted in Sweden. | Fredriksson K. et al. | 2005 | Occupational Ergonomics - Volume 5, Issue 2, pp. 79-88 | Social support or spousal response not included |
| Effects of mindfulness-oriented recovery enhancement versus social support on negative affective interference during inhibitory control among opioid-treated chronic pain patients: A pilot mechanistic study | Garland E. et al. | 2019 | Annals of Behavioral Medicine - Volume 53, Issue 10, pp. 865-876 | Outcomes not evaluated |
| Health and suffering are associated with social support: a cross-sectional study of women and mothers with exhaustion and pain | Gebhardt A. et al. | 2021 | BMC Women's Health - Volume 21, Issue 1, pp | Outcomes not evaluated |
| The role of stress transactional theory on the development of fibromyalgia: a structural equation model. | González-Ramírez M.T. et al. | 2011 | Actas espanolas de psiquiatria - Volume 39, Issue 2, pp. 81-7 | Outcomes not evaluated |
| Chronic neuropathic pain in spinal cord injury patients: What is the impact of social and environmental factors on care management? | Goossens D. et al. | 2009 | Ann. Phys. Rehabil. Med. - Volume 52, Issue 2, pp. 173-179 | Conference abstract |
| Community Fibromyalgia Support Groups: Who do they serve and could they improve outcomes? | Gregory W.J. et al. | 2021 | Physiotherapy - Volume 113, Issue 0, pp. e172 | Conference abstract |
| Impact of a comprehensive lifestyle intervention on pain, depressed mood, stress, inflammation, and telomeric activity for individuals with chronic pain | Hamilton K. et al. | 2019 | Dissertation Abstracts International: Section B: The Sciences and Engineering - Volume 0, Issue 0, pp. | Doctoral thesis |
| One day at a time: The impact of daily satisfaction with spouse responses on pain, negative affect and catastrophizing among individuals with rheumatoid arthritis | Holtzman S. et al. | 2007 | Pain - Volume 131, Issue 1, pp. 202-213 | Social support or spousal response not included |
| The role of social support in coping with daily pain among patients with rheumatoid arthritis | Holtzman S. et al. | 2004 | Journal of Health Psychology - Volume 9, Issue 5, pp. 677-695 | Not chronic pain population |
| Psychosocial Risk and Resilience Factors Associated with Pain in Young Adults | Hong S. et al. | 2024 | J. Pain - Volume 25, Issue 4, pp. 62 | Not retrievable |
| The role of spousal relationships in fibromyalgia patients' quality of life. | Huang E.R. et al. | 2018 | Psychology, health & medicine - Volume 23, Issue 8, pp. 987-995 | Social support or spousal response not included |
| Pain among women: Associations with socio-economic and work conditions | Jablonska B. | 2006 | European Journal of Pain - Volume 10, Issue 5, pp. 435 | Not chronic pain population |
| Social support reduces the impact of chronic pain in individuals with physical disability: A longitudinal study | Jensen M. et al. | 2018 | Mult. Scler. J. - Volume 24, Issue 2, pp. 151 | Conference abstract |
| Musculoskeletal pain and its socioeconomic implications. | Katz W.A. | 2002 | Clinical rheumatology - Volume 21, Issue , pp. S2-4 | Conference abstract |
| Effects of disease activity, illness perception, social support and coping methods on quality of life in the patients with rheumatoid arthritis and fibromyalgia syndrome | Kesmen H. et al. | 2019 | Ann. Rheum. Dis. - Volume 78, Issue 0, pp. 679 | Conference abstract |
| Social support works better in rheumatoid arthritis than fibromyalgia | Kesmen H. et al. | 2020 | Ann. Rheum. Dis. - Volume 79, Issue 0, pp. 913 | Conference abstract |
| Cognitive-behavioral classifications of chronic pain in patients with multiple sclerosis. | Khan F. et al | 2011 | International journal of rehabilitation research. Internationale Zeitschrift fur Rehabilitationsforschung. Revue internationale de recherches de readaptation - Volume 34, Issue 3, pp. 235-42 | Outcomes not evaluated |
| Association of pain, social support and socioeconomic indicators in patients with spinal cord injury in Iran | Khazaeipour Z. et al. | 2017 | Spinal Cord - Volume 55, Issue 2, pp. 180-186 | Data could not be extracted |
| Relationship between pain, social support and socio-economic indicators in individuals with spinal cord injury | Khazaeipour Z. et al. | 2017 | Arch. Phys. Med. Rehabil. - Volume 98, Issue 10, pp. e2 | Conference abstract |
| Predictors of a favourable outcome in patients with fibromyalgia: Results of 1-year follow-up | Kim J.-E. et al. | 2016 | Clinical and Experimental Rheumatology - Volume 34, Issue 3, pp. 521-526 | Social support or spousal response not included |
| Protective and Risk Factors at the Intersection of Chronic Pain, Depression, Anxiety, and Somatic Amplification: A Latent Profile Approach | Kim S. et al. | 2022 | Journal of Pain Research - Volume 15, Issue 0, pp. 1107-1121 | Not chronic pain population |
| Psychosocial factors discriminate multidimensional clinical groups of chronic low back pain patients. | Klapow, J.C. et al. | 1995 | PAIN - Volume 62, Issue 3, pp. 349-355 | Outcomes not evaluated |
| Psycho-social factors and coping strategies as predictors of chronic evolution and quality of life in patients with low back pain: A prospective study | Koleck M. et al. | 2006 | Eur. J. Pain - Volume 10, Issue 1, pp. 1 | Not chronic pain population |
| Social support and invalidation by others contribute uniquely to the understanding of physical and mental health of patients with rheumatic diseases | Kool M.B. et al. | 2013 | Journal of Health Psychology - Volume 18, Issue 1, pp. 86-95 | Not chronic pain population |
| Loneliness in patients with rheumatic diseases: the significance of invalidation and lack of social support. | Kool M.B. et al. | 2012 | The Journal of psychology - Volume 146, Issue 1, pp. 229-41 | Not chronic pain population |
| The association of perceived partner-related social support with self-reported outcomes in women post-mastectomy | Kudel I. et al. | 2008 | Journal of Health Psychology - Volume 13, Issue 8, pp. 1030-1039 | Not chronic pain population |
| Exploring the Multidimensional Nature of Resilience in Pain and Function among Aging Adults with Chronic Low Back Pain | Lakdawala M. | 2021 | J. Pain - Volume 22, Issue 5, pp. 590 | Conference article |
| The role of neuroticism and social support in older adults with chronic pain behavior | Lauver S.C. et al. | 1997 | Personality and Individual Differences - Volume 23, Issue 1, pp. 165-167 | Outcomes not evaluated |
| Social support and cognitive functioning as resources for elderly persons with chronic arthritis pain. | Lee J.E. et al. | 2016 | Aging & Mental Health - Volume 20, Issue 4, pp. 370-379 | Not chronic pain population |
| [Chronic pain and associated factors amongst institutionalized elderly with arthritis]. | Lin J.I. et al. | 2011 | Hu li za zhi The journal of nursing - Volume 58, Issue 1, pp. 59-67 | Not retrievable |
| Alexithymia, social support and health problems | Lumley M.A. et al. | 1996 | Journal of Psychosomatic Research - Volume 41, Issue 6, pp. 519-530 | Not chronic pain population |
| Cross-Sectional Study of Young Adults Diagnosed With Juvenile Fibromyalgia: Social Support and Its Impact on Functioning and Mood. | Lynch-Jordan A.M. et al. | 2015 | The Journal of adolescent health : official publication of the Society for Adolescent Medicine - Volume 57, Issue 5, pp. 482-7 | Paediatric population |
| The moderating effects of social support and depressive symptoms on pain among elderly multimorbid patients—data from the multicentre, prospective, observational cohort study MultiCare. | Mallon T.S. et al. | 2022 | Aging & Mental Health - Volume 26, Issue 4, pp. 803-809 | Not chronic pain population |
| Chronic pain management and social supporting in older people: A qualitative study | Manoochehri H. et al. | 2014 | Advances in Environmental Biology - Volume 8, Issue 6, pp. 3098-3105 | Outcomes not evaluated |
| Comorbidity of chronic back pain and depression in Germany: Results from the GEDA study, 2009 and 2010. | Martini L. et al. | 2018 | Zeitschrift fur Evidenz, Fortbildung und Qualitat im Gesundheitswesen - Volume 137, Issue , pp. 62-68 | Outcomes not evaluated |
| The association between severity of dysmenorrhea and social support among female students of ilam university of medical sciences | Mashreghi M. et al. | 2021 | Shiraz E Medical Journal - Volume 22, Issue 9, pp. | Not chronic pain population |
| Buffer or amplifier? Longitudinal effects of social support for functional autonomy/dependence on older adults' chronic pain experiences | Matos M. et al. | 2017 | Health Psychology - Volume 36, Issue 12, pp. 1195-1206 | Not retrievable |
| Social context and acceptance of chronic pain: The role of solicitous and punishing | McCracken L.M. | 2005 | Pain - Volume 113, Issue 1, pp. 155-159 | Not chronic pain population |
| Interpersonal Responses and Pain Management Within the US Military | McGeary C.A. et al. | 2016 | Journal of occupational rehabilitation - Volume 26, Issue 2, pp. 216‐228 | Outcomes not evaluated |
| The relation of social support and depression in patients with chronic low back pain | McKillop A.B. et al. | 2017 | Disability and Rehabilitation - Volume 39, Issue 15, pp. 1482-1488 | Not chronic pain population |
| The Impact of the Quantity and Quality of Social Support on Patients with Chronic Pain. | McMurtry M. et al. | 2020 | Current Pain & Headache Reports - Volume 24, Issue 11, pp. N.PAG-N.PAG | Data could not be extracted |
| Longitudinal patterns of pain in patients with diffuse and limited systemic sclerosis: integrating medical, psychological, and social characteristics | Merz E.L. et al. | 2017 | Qual. Life Res. - Volume 26, Issue 1, pp. 85-94 | Not chronic pain population |
| A Comprehensive Pain-Related Risk And Protective Index And Machine Learning Brain Age Gap | Mickle A, et al. | 2024 | J. Pain - Volume 25, Issue 4, pp. 23 | Not retrievable |
| The Role of Resilience, Happiness, and Social Support in the Psychological Function during the Late Stages of the Lockdown in Individuals with and without Chronic Pain. | Miró J. et al. | 2022 | International journal of environmental research and public health - Volume 19, Issue 11, pp. | Outcomes not evaluated |
| Influence of social support and emotional context on pain processing and magnetic brain responses in fibromyalgia | Montoya P. et al. | 2004 | Arthritis Rheum. - Volume 50, Issue 12, pp. 4035-4044 | Outcomes not evaluated |
| Cancer patients with pain: The spouse/partner relationship and quality of life | Morgan M.A. et al. | 2011 | Cancer Nursing - Volume 34, Issue 1, pp. 13-23 | Not chronic pain population |
| Social Contribution and Psychological Well-Being among Midlife Adults with Chronic Pain: A Longitudinal Approach | Nguyen N.P. et al. | 2020 | J Aging Health - Volume 32, Issue 10, pp. 1591-1601 | Not chronic pain population |
| Depressive symptoms in patients with chronic pain. | Nicholas M.K. et al. | 2009 | The Medical journal of Australia - Volume 190, Issue 0, pp. S66-70 | Not chronic pain population |
| Long-term functioning following whiplash injury: The role of social support and personality traits | Nijs J. et al. | 2011 | Clinical Rheumatology - Volume 30, Issue 7, pp. 927-935 | Not chronic pain population |
| Perceived social support is strongly associated with recovery after injury | Orlas C.P. et al. | 2021 | Journal of Trauma and Acute Care Surgery - Volume 91, Issue 3, pp. 552-558 | Not retrievable |
| Psychosocial factors associated with pain intensity, pain-related interference, and psychological functioning in persons with multiple sclerosis and pain. | Osborne T.L. et al. | 2007 | Pain - Volume 127, Issue 1, pp. 52-62 | Not chronic pain population |
| Quality of Life among Lower Limb Amputees with Chronic Phantom Limb Pain or Stump Pain at a Tertiary Institution's Pain Clinic | Pasutharnchat K. et al. | 2022 | J. Med. Assoc. Thailand - Volume 105, Issue 3, pp. 228-239 | Not retrievable |
| Prevalence of fibromyalgia in medical students and its association with lifestyle factors - a cross-sectional study | Patel A. et al. | 2021 | Reumatologia - Volume 59, Issue 3, pp. 138-145 | Social support or spousal response not included |
| Social support and functional status in chronic pain patients | Patrick L. et al. | 1996 | Canadian Journal of Rehabilitation - Volume 9, Issue 4, pp. 195-201 | Not retrievable |
| The effects of perceived versus enacted social support on the discriminative cue function of spouses for pain behaviors. | Paulsen J.S. et al. | 1995 | Pain - Volume 60, Issue 1, pp. 103-110 | Outcomes not evaluated |
| Pain related variables associated with perceived spouse responses to patient pain and well behavior | Pence L.B. et al. | 2009 | J. Pain - Volume 10, Issue 4, pp. S63 | Conference abstract |
| Social support buffers the negative influence of injustice perceptions on pain interference in persons living with HIV (PLWH) and chronic pain | Penn T. et al. | 2018 | J. Pain - Volume 19, Issue 3, pp. S59 | Outcomes not evaluated |
| The efficacy of a novel facebook-based psychosocial intervention for adults with chronic pain: A randomized clinical trial | Pester B.D. et al. | 2021 | Dissertation Abstracts International: Section B: The Sciences and Engineering - Volume 0, Issue 0, pp | Doctoral thesis |
| Structural equation modeling of disability in women with fibromyalgia or multiple sclerosis | Phillips L.J. et al. | 2009 | West. J. Nurs. Res. - Volume 31, Issue 1, pp. 89-109 | Not chronic pain population |
| Social Relationship Quality Among Patients With Chronic Pain: A Population-Based Sample. | Philpot L.M. et al. | 2020 | Journal of patient experience - Volume 7, Issue 3, pp. 316-323 | Not chronic pain population |
| Significant other interactions in people with chronic low back pain: Subgrouping and multidimensional profiles | Rabey M. et al. | 2022 | Br. J. Pain - Volume 16, Issue 3, pp. 326-340 | Outcomes not evaluated |
| Cognitions, Coping, and Social Environment Predict Adjustment to Pain in Spinal Cord Injury | Raichle K.A. et al. | 2007 | J. Pain - Volume 8, Issue 9, pp. 718-729 | Not chronic pain population |
| An application of the biopsychosocial model for pain in Canadian Veterans Life After Service Studies 2019 survey. | Reyes-Vélez J. et al. | 2024 | Journal of Military, Veteran & Family Health - Volume 10, Issue 1, pp. 30-39 | Not chronic pain population |
| Predicting consistency of pain over a 10-year period in persons with spinal cord injury | Rintala D.H. et al. | 2004 | Journal of Rehabilitation Research and Development - Volume 41, Issue 1, pp. 75-88 | Not retrievable |
| Does social support impact on exercise adherence in patients with chronic low back pain? | Roberts K. Et al. | 2023 | J. Sci. Med. Sport - Volume 26, Issue 0, pp. S171-S172 | Conference article |
| Reported Pain and Fatigue Behaviors Mediate the Relationship Between Catastrophizing and Perceptions of Solicitousness in Patients With Chronic Fatigue. | Romano J.M. et al. | 2016 | Journal of Pain - Volume 17, Issue 3, pp. 328-335 | Not chronic pain population |
| Relationship satisfaction moderates the associations between male partner responses and depression in women with vulvodynia: A dyadic daily experience study | Rosen N.O. et al. | 2014 | Pain - Volume 155, Issue 7, pp. 1374-1383 | Not chronic pain population |
| Risk factors for fibromyalgia: the role of violence against women. | Ruiz-Pérez I. et al. | 2009 | Clinical rheumatology - Volume 28, Issue 7, pp. 777-86 | Outcomes not evaluated |
| Pain intensity, temperament traits and social support as determinants of trauma symptoms in patients suffering from rheumatoid arthritis and low-back pain | Rzeszutek M. et al. | 2016 | Int. J. Rheum. Dis. - Volume 19, Issue 4, pp. 412-419 | Outcomes not evaluated |
| Evaluation of clinical and social factors in fibromyalgia syndrome | Salgueiro M. et al. | 2009 | Valoración de factores sociales y clínicos en el síndrome de fibromialgia - Volume 16, Issue 6, pp. 323-329 | Article in foreign language |
| Does Self-compassion Benefit Couples Coping With Vulvodynia? Associations With Psychological, Sexual, and Relationship Adjustment. | Santerre-Baillargeon M. et al. | 2018 | Clinical Journal of Pain - Volume 34, Issue 7, pp. 629-637 | Not chronic pain population |
| Social Support Is Inversely Associated With Sleep Disturbance, Inflammation, and Pain Severity in Chronic Low Back Pain | Saravanan A. et al. | 2021 | Nursing research - Volume 70, Issue 6, pp. 425-432 | Not retrievable |
| Relative contributions of spousal support and illness appraisals to depressed mood in arthritis patients. | Schiaffino K.M. et al. | 1995 | Arthritis care and research : the official journal of the Arthritis Health Professions Association - Volume 8, Issue 2, pp. 80-7 | Not chronic pain population |
| Longitudinal outcomes associated with significant other responses to chronic fatigue and pain. | Schmaling K.B. et al. | 2020 | Journal of health psychology - Volume 25, Issue 5, pp. 692-702 | Not chronic pain population |
| Death of a lifestyle: the effects of social support and healthcare support on the quality of life of persons with fibromyalgia and/or chronic fatigue syndrome. | Schoofs N. et al. | 2004 | Orthopedic nursing - Volume 23, Issue 6, pp. 364-74 | Not chronic pain population |
| Psychosocial factors associated with poor outcomes after amputation for complex regional pain syndrome type-I | Schrier E. et al. | 2019 | PLoS ONE - Volume 14, Issue 3, pp. | Outcomes not evaluated |
| An Examination of the Disablement Process among Older American Indians: The Native Elder Care Study | Schure M.B. et al. | 2016 | Gerontologist - Volume 56, Issue 5, pp. 948-955 | Not chronic pain population |
| Pain experience and social support of endometriosis patients during the COVID-19 pandemic in Germany – Results of a web-based cross-sectional survey | Schwab R. et al. | 2021 | PLoS ONE - Volume 16, Issue 8, pp. | Not chronic pain population |
| High prevalence of depression and anxiety in patients with endometriosis during the SARS-CoV-2 pandemic in Germany | Schwab R. et al. | 2021 | Hum. Reprod. - Volume 36, Issue 0, pp. i271 | Conference article |
| Mental Health and Social Support Are Key Predictors of Resilience in German Women with Endometriosis during the COVID-19 Pandemic | Schwab R. et al. | 2022 | J. Clin. Med. - Volume 11, Issue 13, pp. | Not chronic pain population |
| The role of pain, disability and perceived social support in psychological and academic functioning of university students with pain: An observational study | Serbic D. et al. | 2021 | International Journal of Adolescent Medicine and Health - Volume 33, Issue 3, pp. 209-217 | Data could not be extracted |
| Childhood trauma among women with chronic pelvic pain and women with chronic back pain: Relationship between type of trauma and site of pain | Simon J.I. | 1997 | Dissertation Abstracts International: Section B: The Sciences and Engineering - Volume 0, Issue 0, pp. 2741 | Doctoral thesis |
| Psychological Factors Influencing Results of Cholecystectomy | Stefaniak T. et al. | 2004 | Scandinavian Journal of Gastroenterology - Volume 39, Issue 2, pp. 127-132 | Data could not be extracted |
| Emotional Support, Post-Traumatic Stress Disorder and Risk for Long-Term, High-Dose, Daily Prescription Opioid Use | Sullivan M. Et al. | 2024 | J. Pain - Volume 25, Issue 4, pp. 60 | Not retrievable |
| Rates of physical activity and perceived social support among young adult women with juvenile-onset fibromyalgia. | Suorsa K. et al. | 2016 | Journal of Pain - Volume 17, Issue 0, pp. S100-S101 | Conference article |
| Decreasing prevalence of chronic back pain in Catalonia. Analysis of the Catalan Health Survey. | Surís X. et al. | 2022 | Public Health (Elsevier) - Volume 206, Issue 0, pp. 38-45 | Not retrievable |
| The impact of chronic widespread pain on health status and long-term health predictors: a general population cohort study. | Sylwander C. et al. | 2020 | BMC musculoskeletal disorders - Volume 21, Issue 1, pp. 36 | Outcomes not evaluated |
| Relationship status and quality moderate daily pain-related changes in physical disability, affect, and cognitions in women with chronic pain | Taylor S.S. et al. | 2013 | Pain - Volume 154, Issue 1, pp. 147-153 | Outcomes not evaluated |
| Chronic pain and depression: Is social support relevant? | Trief P.M. et al. | 1995 | Psychological Reports - Volume 76, Issue 1, pp. 227-236 | Outcomes not evaluated |
| Predictors of distress and depression in elders with arthritic pain. | Tsai P.F. et al. | 2005 | Journal of advanced nursing - Volume 51, Issue 2, pp. 158-65 | Not chronic pain population |
| Longitudinal relationships between perceived social support and quality-of-life among patients with chronic pain | Van Dyke B. et al. | 2018 | J. Pain - Volume 19, Issue 3, pp. S25 | Conference article |
| Risk factors for longer term psychological distress in well-functioning fibromyalgia patients: A prospective study into prognostic factors | van Koulil S. et al. | 2010 | Patient Education and Counseling - Volume 80, Issue 1, pp. 126-129 | Outcomes not evaluated |
| Social support modifies association between forward bending of the trunk and low-back pain: Cross-sectional field study of blue-collar workers | Villumsen M. et al. | 2016 | Scand. J. Work Environ. Health - Volume 42, Issue 2, pp. 125-134 | Not chronic pain population |
| Social support for women with chronic pelvic pain: what is helpful from whom? | Warwick R. et al. | 2004 | Psychology & Health - Volume 19, Issue 1, pp. 117-134 | Outcomes not evaluated |
| [Influence of social support and personality traits on psychological characteristic of patients with chronic cervicodynia and lumbodynia]. | Wei J. et al. | 2012 | Zhongguo gu shang = China journal of orthopaedics and traumatology - Volume 25, Issue 3, pp. 216-9 | Not retrievable |
| Relationship between pain characteristics and pain adaptation type in persons with SCI. | Widerström-Noga E.G. et al, | 2009 | Journal of Rehabilitation Research & Development - Volume 46, Issue 1, pp. 43-56 | Data could not be extracted |
| The psycho-social dimension of pain and health-related quality of life in the oldest old. | Willman A. et al. | 2013 | Scandinavian Journal of Caring Sciences - Volume 27, Issue 3, pp. 534-540 | Not chronic pain population |
| Profiles of Risk and Resilience in Chronic Pain: Loneliness, Social Support, Mindfulness, and Optimism Coming out of the First Pandemic Year | Wilson J.M. et al. | 2022 | Pain Med. - Volume 23, Issue 12, pp. 2010-2021 | Outcomes not evaluated |
| Daily Spousal Responsiveness Predicts Longer-Term Trajectories of Patients' Physical Function | Wilson S.J. et al. | 2017 | Psychol Sci - Volume 28, Issue 6, pp. 786-797 | Outcomes not evaluated |
| Influence of work-related psychosocial factors on the prevalence of chronic pain and quality of life in patients with chronic pain. | Yamada K. et al. | 2016 | BMJ open - Volume 6, Issue 4, pp. e010356 | Outcomes not evaluated |
| Predictors of chronic pelvic pain in female population | Zagorulko O. et al. | 2023 | Rus. J. Pain. - Volume 21, Issue 2, pp. 58-65 | Article in foreign language |
| Social support and pain among persons with spinal cord injury | Zheng J. et al. | 2019 | Psychosom. Med. - Volume 81, Issue 4, pp. A57-A58 | Not retrievable |
| Prediction of Chronic Muscular Pain Based on Personality Type, Hardiness and Perceived Social Support | Zoghipaydar M.R. et al. | 2020 | Sadra Med. Sci. J. - Volume 8, Issue 4, pp | Article in foreign language |

*Reason for exclusion (explanation):*

1. ***Outcomes not evaluated****: studies that did not evaluate the link between any of the three social support measures and at least one of our study outcomes.*
2. ***Social support or spousal response not included****: studies that did not include any form of perceived social support, social support satisfaction or spousal responses in their study.*
3. ***Not chronic pain population****: studies that did not include chronic pain patients according to our inclusion criteria.*
4. ***Paediatric population****: studies that included paediatric patients.*
5. ***Data could not be extracted****: studies from which data could not be extracted due to the specificity of the model in the study.*
6. ***Conference abstracts****: articles that were published only as conference abstracts.*
7. ***Doctoral thesis****: abstracts that were linked to a doctoral thesis.*
8. ***Article in foreign language****: articles that weren’t in English, French or Italian.*
9. ***Not retrievable****: articles that could not be retrieved.*
