# Supplementary material for: Navigating the biopsychosocial landscape: A systematic review on the association between social support and chronic pain
Source: PLoS One. 2025 Apr 29;20(4):e0321750. doi: 10.1371/journal.pone.0321750 (PMC12040255; doi:10.1371/journal.pone.0321750)
Supplement: S3 Table — PSS: perceived social support, 0: no correlation. 0: no relationship., −: negative correlation. ⊝: negative correlation, but no significantly negative relationship found. −: negative relationship., +: positive correlation. ⊕: positive correlation, but no significantly positive relationship found. +: positive relationship. (DOCX) [file pone.0321750.s006.docx]

**S3 Table. Impact of PSS in screened studies that were excluded from the final analysis**

| STUDY | **Bakers A. et al., 2011** | **Bergmans S. et al., 2001** | **Cano A., 2004** | **Donaghy B. et al., 2022** | **Du Plessis M., 2009** | **Evers A. et al., 2003** | **Faucett J.A. et al., 1991** | | **Freitas R.P.A. et al., 2017** | **Gunduz N. et al., 2019** | **Jensen M.P. et al., 2002** | **Larbig W, et al., 2019** | **Lee G.K. et al., 2007** | **Lopez-Martinez A.E. et al, 2008** | **Muramatsu N. et al., 1997** | **Phillips L.J. et al., 2010** | | **Zeng F. et al., 2016** | **No. of reports on this factor** | **Negative association** | **No association** | **Positive association** |
| --- | --- | --- | --- | --- | --- | --- | --- | --- | --- | --- | --- | --- | --- | --- | --- | --- | --- | --- | --- | --- | --- | --- |
| TYPE OF  SOCIAL  SUPPORT | PSS – Emotional, instrumental and informational | PSS – *Personal support* | PSS | PSS – Instrumental | PSS | PSS – Emotional and instrumental | PSS – Emotional | | PSS – Emotional, instrumental and informational | PSS | PSS | PSS | PSS | PSS - Emotional | PSS – Emotional and instrumental | PSS - Emotional | PSS | PSS – Emotional, instrumental and informational |  |  |  |  |
| Sample Size  Variable | N = 247 | N = 1852 | N = 96 | N = 86 | N = 32 | N = 78 | N = 84 | N = 67 | N = 17 | N = 65 | N = 61 | N = 51 | N = 171 | N = 117 | N = 1691 | N = 118 | N = 197 | N = 147 |  | **−** | 0 | **+** |
| Pain Intensity |  |  | − | **0** | 0 | **−** | 0 | 0 | − | − | 0 |  |  | **−** |  |  |  |  | 10 | 5 | 5 | 0 |
| Pain development | ⊕ | **−** |  |  |  |  |  |  |  |  |  | + |  |  | + |  |  |  | 4 | 1 | 0 | 3 |
| Pain interference/  disability |  |  |  | **0** |  | **−** |  |  | 0 |  | **−** |  |  | **0** |  | **0** | ⊝ |  | 7 | 3 | 4 | 0 |
| QoL |  |  |  |  |  |  |  |  | 0 |  |  |  |  |  |  |  |  |  | 1 | 0 | 1 | 0 |
| Physical QoL |  |  |  |  |  |  |  |  |  |  |  |  |  |  |  |  |  |  | 0 | 0 | 0 | 0 |
| Mental QoL |  |  |  |  |  |  |  |  |  |  |  |  |  |  |  |  |  |  | 0 | 0 | 0 | 0 |
| Depression | **0** |  |  |  |  |  |  |  | − | − | **−** |  | **−** | **−** |  | **−** | **−** |  | 8 | 7 | 1 | 0 |
| Anxiety |  |  |  |  |  |  |  |  | 0 | − |  |  |  |  |  |  |  | ⊝ | 3 | 2 | 1 | 0 |
