# Supplementary material for: Navigating the biopsychosocial landscape: A systematic review on the association between social support and chronic pain
Source: PLoS One. 2025 Apr 29;20(4):e0321750. doi: 10.1371/journal.pone.0321750 (PMC12040255; doi:10.1371/journal.pone.0321750)
Supplement: S4 Table — SSS: social support satisfaction, 0: no correlation. 0: no relationship., −: negative correlation. ⊝: negative correlation, but no significantly negative relationship found. −: negative relationship., +: positive correlation. ⊕: positive correlation, but no significantly positive relationship found. +: positive relationship. (DOCX) [file pone.0321750.s007.docx]

**S4 Table. Impact of SSS in screened studies that were excluded from the final analysis**

| STUDY | **Kerns R.D. et al, 1990** | **Leonard M.T. et al, 2018** | **Reich J.W. et al., 2006** | | **Saarijärvi S. et al., 1990** | | **No. of studies on this factor** | **Negative association** | **No association** | **Positive association** |
| --- | --- | --- | --- | --- | --- | --- | --- | --- | --- | --- |
| TYPE OF SSS | Global marital satisfaction | Relationship satisfaction | Relationship satisfaction | | Marital satisfaction | |  |  |  |  |
| Sample Size  Variable | N = 106 | N = 78 | N = 51 | N = 32 | N = 31 | N = 32 |  | **-** | **/** | **+** |
| Pain Intensity | **0** |  | - | 0 |  |  | 3 | 1 | 2 | 0 |
| Pain interference/  disability |  |  | - | 0 |  |  | 2 | 1 | 1 | 0 |
| QoL |  |  |  |  |  |  | 0 | 0 | 0 | 0 |
| Physical QoL |  |  |  |  |  |  | 0 | 0 | 0 | 0 |
| Mental QoL |  |  |  |  |  |  | 0 | 0 | 0 | 0 |
| Depression | **-** | - |  |  | 0 | - | 4 | 3 | 1 | 0 |
| Anxiety |  |  |  |  | - | - | 2 | 2 | 0 | 0 |
