# Supplementary material for: Navigating the biopsychosocial landscape: A systematic review on the association between social support and chronic pain
Source: PLoS One. 2025 Apr 29;20(4):e0321750. doi: 10.1371/journal.pone.0321750 (PMC12040255; doi:10.1371/journal.pone.0321750)
Supplement: S5 Table — 0: no correlation. 0: no relationship., −: negative correlation. ⊝: negative correlation, but no significantly negative relationship found. −: negative relationship., +: positive correlation. ⊕: positive correlation, but no significantly positive relationship found. +: positive relationship. (DOCX) [file pone.0321750.s008.docx]

**S5 Table. Impact of spousal responses in screened studies that were excluded from final analysis**

| STUDY | **Buenaver L. Et al., 2006** | | | **Cano A. et al., 2000** | | | **Cano A., 2004** | | | | **Faucett J.A. et al., 1991** | | | | **Ginting J.V. et al., 2011** | | | **Jensen M.P. et al., 2002** | **Kerns R.D. et al., 1990** | | | **Leonard M.T. et al, 2018** | | | **Nees F. et al., 2022** | | **Oraison H.M. et al., 2021** | **Reich J.W. et al., 2006** | | **Turk D.C. et al., 1992** | | **No. of reports on this factor** | **Negative association** | **No association** | **Positive association** |
| --- | --- | --- | --- | --- | --- | --- | --- | --- | --- | --- | --- | --- | --- | --- | --- | --- | --- | --- | --- | --- | --- | --- | --- | --- | --- | --- | --- | --- | --- | --- | --- | --- | --- | --- | --- |
| TYPE OF  SPOUSAL  RESPONSES | Perceived spousal support | Perceived negative responses | perceived solicitous responses | Perceived negative responses | perceived solicitous responses | Perceived distracting responses | Perceived spousal support | Perceived negative responses | perceived solicitous responses | Perceived distracting responses | Perceived negative responses | | perceived solicitous responses | | Perceived negative responses | perceived solicitous responses | Perceived distracting responses | perceived solicitous responses | Perceived negative responses | perceived solicitous responses | Perceived distracting responses | Perceived negative responses | perceived solicitous responses | Perceived distracting responses | Solicitous spouse | Non- solicitous spouse | Perceived spousal support | Perceived spousal support | | Perceived negative responses | perceived solicitous responses |  |  |  |  |
| Sample Size  Variable | N = 1365 | N = 1365 | N = 1365 | N = 165 | N = 165 | N = 165 | N = 96 | N = 96 | N = 96 | N = 96 | N = 84 | N = 67 | N = 84 | N = 67 | N = 188 | N = 188 | N = 188 | N = 61 | N = 106 | N = 106 | N = 106 | N = 78 | N = 78 | N = 78 | N = 10 | N = 10 | N = 201 | N = 51 | N = 32 | N = 148 | N = 148 |  | **-** | **/** | **+** |
| Pain Intensity | + | + | + | **+** | 0 | 0 | - | + | 0 | 0 | **-** | 0 | 0 | 0 | 0 | + | + | 0 | 0 | + | + |  |  |  | **+** | **0** | - | 0 | 0 | 0 | + | 28 | 3 | 14 | 11 |
| Pain interference/  disability | + | + | + |  |  |  |  |  |  |  |  |  |  |  | + | + | + | **+** |  |  |  |  |  |  |  |  | **-** | 0 | - |  |  | 10 | 2 | 0 | 7 |
| QoL |  |  |  |  |  |  |  |  |  |  |  |  |  |  |  |  |  |  |  |  |  |  |  |  |  |  |  |  |  |  |  | 0 | 0 | 0 | 0 |
| Physical QoL |  |  |  |  |  |  |  |  |  |  |  |  |  |  | - | - | - |  |  |  |  |  |  |  |  |  |  |  |  |  |  | 3 | 3 | 0 | 0 |
| Mental QoL |  |  |  |  |  |  |  |  |  |  |  |  |  |  | - | 0 | 0 |  |  |  |  |  |  |  |  |  |  |  |  |  |  | 3 | 1 | 2 | 0 |
| Depression | 0 | + | 0 | ⊕ | 0 | 0 |  |  |  |  |  |  |  |  | + | 0 | 0 | **+** | + | 0 | 0 | - | 0 | 0 |  |  |  |  |  |  |  | 16 | 1 | 10 | 5 |
| Anxiety |  |  |  |  |  |  |  |  |  |  |  |  |  |  |  |  |  |  |  |  |  |  |  |  |  |  |  |  |  |  |  | 0 | 0 | 0 | 0 |
