# Supplementary material for: Navigating the biopsychosocial landscape: A systematic review on the association between social support and chronic pain
Source: PLoS One. 2025 Apr 29;20(4):e0321750. doi: 10.1371/journal.pone.0321750 (PMC12040255; doi:10.1371/journal.pone.0321750)
Supplement: Attachment — Submitted filename: PLOS ONE - Rebuttal letter.docx [file pone.0321750.s010.docx]

**Replies to Reviewers**

| Reviewer #1 | |
| --- | --- |
| Comment 1:  “The excluded full-text reports need to be presented in a supplementary list. However, some modifications are needed as some reports should have been excluded in the title and abstract phase.” | “Annex 6” has been added to the study, where we report the list of the full-text articles that have been excluded along with the reason for exclusion. Literature Review articles (Bernardes S. et al., 2017 ; Cooper K. et al., 2013 ; Zaza C. et al., 2002) could have been excluded based on the title and abstract. This has been corrected in the revised manuscript. |
| Comment 2:  “Assessing the quality of evidence using the GRADE is not very well matching this guideline in specific. We suggest either giving more details in the methods section about the tool and the table you created.” | Our manuscript acknowledges that we did not use the GRADE system, as it was not well suited to this study. Instead, we used an adapted version of the Quality Assessment Tool by Hawker et al., as detailed in the Materials and Methods, section 2.5 (lines 209-222) and Annex 3. |
| Reviewer #2 | |
| Abstract: Lacks clarity and not compressive | With no additional information, we cannot address the comment on “clarity, and we do not understand what is meant by the term “not compressive”. |
| Background: Lacks strength and focus as well as smooth transition | With no additional information, we cannot address this vague statement on the lack of “strength and focus” or a “smooth transmission”.  We would like to highlight the fact that, despite these negative comments, Reviewer #2 nevertheless replied yes to the following two statements. First that the manuscript “*describes a technically sound piece of scientific research with data that supports the conclusions. Experiments must have been conducted rigorously, with appropriate controls, replication, and sample sizes. The conclusions must be drawn appropriately based on the data presented”. Second, that* “the manuscript (is) presented in an intelligible fashion and written in standard English?”. |
| Methods: Incomplete and the dependent variable should be well operationalized | With no additional information, we cannot address the statement that results are “incomplete”. Also, we do not understand the following sentence “… the dependent variable should be well operationalized”. The latter negative comment on the dependent variable, appears to contradict the positive answer to point 2: *“Has the statistical analysis been performed appropriately and rigorously?”* |
| Results and discussion: Inadequately clear, brief and logical. The discussion lacks consistency throughout its contents and proper explanation and references.” | How can this section be “inadequately clear”? We would also need further information on how the “discussion lacks consistency” and which “explanation and references” the reviewer is referring to.  Overall, the comments of the 2nd reviewer were extremely succinct and vague to the point that we question whether he/she read our work. Moreover, they are contradictory to those of Reviewer #1. |
