# Supplementary material for: Navigating the biopsychosocial landscape: A systematic review on the association between social support and chronic pain
Source: PLoS One. 2025 Apr 29;20(4):e0321750. doi: 10.1371/journal.pone.0321750 (PMC12040255; doi:10.1371/journal.pone.0321750)
Supplement: Attachment — Submitted filename: Response to Reviewers.docx [file pone.0321750.s011.docx]

**Journal requirements**

| **Comment** | **Answer** |
| --- | --- |
| **1.** **Please ensure that your manuscript meets PLOS ONE's style requirements, including those for file naming.** | We adapted our manuscript as required by the Plos One’s style requirements.  Changes are highlighted in the 'Revised Manuscript with Track Changes'. |
| **2. Financial disclosure. Please state what role the funders took in the study.** | Please find below the amended financial disclosure statement:  " This project has received funding from the European Union’s Horizon2020 research and innovation program under grant agreement No 848068. The funders had no role in study design, data collection and analysis, decision to publish, or preparation of the manuscript."  The financial disclosure has been included in the Cover Letter |
| **3. Acknowledgements. We note that you have provided funding information that is currently declared in your Funding Statement. However, funding information should not appear in the Acknowledgments section or other areas of your manuscript. We will only publish funding information present in the Funding Statement section of the online submission form.** | We removed the Funding Statement from the manuscript (Acknowledgements section).  Please find below the amended Funding Statement:  “This project has received funding from the European Union’s Horizon2020 research and innovation program under grant agreement No 848068. This publication reflects only the authors' view and the European Commission is not responsible for any use that may be made of the information it contains.” |
| **4. Please include a separate caption for each figure in your manuscript** | We adapted our manuscript as required by the Plos One’s style requirements.  Changes are highlighted in the 'Revised Manuscript with Track Changes'. |
| **5. Please include your tables as part of your main manuscript and remove the individual files. Please note that supplementary tables (should remain/ be uploaded) as separate "supporting information" files** | We adapted our manuscript as required by the Plos One’s style requirements.  Changes are highlighted in the 'Revised Manuscript with Track Changes'.  We changed the name of the following annexes as follows:   - Annex 1 --> S1 Text - Annex 2 --> S2 Text - Annex 3 --> S3 Text - Annex 4 --> S1 Table - Annex 5 --> S2 Table - Annex 6 --> S3 Table - Annex 7 --> S4 Table - Annex 8 --> S5 Table |

**Responses to reviewers**

| **Reviewer #3:** | |
| --- | --- |
| **Comment** | **Answer** |
| **I would like to see more discussions/future directions on how your findings could translate to social support based treatments/interventions. In my opinion, this is important for the clinical world.** | **Line 723**: “Given the association between perceived social support and better psychological health outcomes, future interventions could target enhancing the psychological aspects of social interactions. Cognitive-behavioural therapy could be modified to include components that focus on improving perceived social support and satisfaction within relationships.  Developing educational programs for both patients and their spouses could help them understand the impact of different support behaviours on pain perception and psychological well-being. Including spouses in treatment sessions may foster better understanding and adaptation, helping them to learn effective ways to support their partner and recognize the boundaries of their involvement.  We also suggest routine screening for psychological distress in patients with chronic pain and evaluation of the quality and type of social support these patients experience. This could lead to a more personalized care approach in future pain counselling.” |
| **Reviewer #4:** | |
| Comment | Answer |
| **Line 33-34: Indicate the Risk of bias tool utilized.** | **Line 32-34**: Out of 11,908 queried results, 67 studies met our inclusion criteria. After assessing for risk of bias (adapted version of the “JBI Critical Appraisal Checklist for Analytical Cross-Sectional Studies”) and quality of evidence (adapted version of the quality assessment tool of Hawker and al.), 35 studies were included in the final analysis. |
| **Line 40: Here you write “ most of the studies were cross-sectional” – in line 211 you write “The studies included in our review are exclusively observational”. Please change the sentences being consistent.** | **Line 219-220**: The studies included in our review predominantly consist of cross-sectional observational studies and are therefore considered of low-quality evidence according to this system. |
| **Line 48: “a survey showed that” … Consider indicating the argument of the survey to facilitate reading** | **Line 47-49**: In 2006, a survey showed that that roughly 60% of individuals with chronic pain reported a reduced capacity or inability **to** work outside their homes, and approximately 20% had experienced job loss due to pain (1). The survey highlights the urgency of improving recognition, treatment, and management of chronic pain within our health system to enhance the quality of life for the affected individuals and reduce economic burdens on our society. |
| **Line 93: point at the end of the sentence.** | **Line 89-91:** It is therefore crucial to clarify which aspects of SS are the most relevant to the aims and context of a specific research question and to define the concept of SS that is used. |
| **Line 130-132: This part has to be moved to the discussion session** | We have moved the following paragraph in the discussion session.  **Line 525-527**: It is important to mention that all the measures of SS in this review are subjective measures from the patient’s point of view. Consequently, such reports are influenced by the way the respondent perceives and processes the information. |
| **Line 133-136: The reference is old. Please specify whether something updated exists or whether this subdivision is still applicable today.** | We’ve added a more recent reference ([30]) from Che X. et al, 2018; at **line 133-139**:  Despite being older theories, a more recent systematic review [30] presented evidence supporting both the main and the buffering effect. However, the buffering effect was more often able to explain findings in studies that were deemed of higher quality. The evidence suggests that pain reduction, on a social level, is partially mediated by the process of support buffering the adverse influences of stress, through processes such as stress appraisal and active coping. We also have to keep in mind that the two theoretical accounts may not be mutually exclusive.  We removed the following sentence (originally at line 156):  « A final comprehensive review led by Che et al. suggested that the buffering effect of SS more often describes the benefits on the pain experience, but it was unable to examine the different types of SS (30). » |
| **Line 139: consider a recent SR (Bisconti M, Esposto M, Tamborrino A, Brindisino F, Giovannico G, Salvioli S. Is Social Support Associated With Clinical Outcomes in Adults With Nonspecific Chronic Low Back Pain? A Systematic Review. Clin J Pain. 2024 Oct 1;40(10):607-617. doi: 10.1097/AJP.0000000000001239. PMID: 39268726.)** | We have included the suggested SR in **line 150-154**: A more recent systematic review [32] analyzed the relationship between social support and clinical outcomes (pain and disability) in individuals with non-specific chronic low back pain. A small association was found between social support and both pain and disability in people with non-specific chronic low back pain. However, due to missing data, it was not possible to analyze differences according to sex, gender, or type of social support. |
| **Line 618: Consider to explain better in method how did you adapt the tool.** | We have added the following sentence to explain how we adapted the tool at **line 686-688**: “We adapted the data analysis question, to make it applicable to all types of studies and not qualitative ones exclusively.” |
